# The Desert Whale: the boom and bust of hemp in Arizona

**DOI:** 10.1186/s42238-023-00187-8

**Published:** 2023-06-09

**Authors:** Anastasia K. Stats, Ken G. Sweat, Robert N. Masson, Kendra D. Conrow, Amy E. Frazier, Maxwell C. K. Leung

**Affiliations:** 1 School of Geographical Sciences & Urban Planning, Tempe, USA; 2 School of Mathematical and Natural Sciences, New College of Interdisciplinary Arts and Sciences, Arizona State University, 4701 W Thunderbird Rd, Glendale, AZ 85306 USA; 3 Cooperative Extension, the University of Arizona, Tucson, USA

**Keywords:** Hemp, Cannabis, Tetrahydrocannabinol (THC), Cannabidiol (CBD), Terpenes, Microgreens, Hempcrete

## Abstract

**Background:**

This paper examines the factors that led to the collapse of hemp grown for cannabidiol (CBD) in Arizona, the United States of America (USA), and particularly in Yuma County, which is a well-established agricultural area in the state.

**Methods:**

This research uses a combination of mapping analysis along with a survey of hemp farmers to assess the reasons why the hemp industry collapsed as well as to foster solutions to these problems.

**Results:**

In 2019, 5430 acres were sown with hemp seed in Arizona with 3890 acres inspected by the state to determine if they could be harvested. By 2021, there were only 156 acres planted, and only 128 of those acres were inspected by the state for compliance. (Crop mortality accounts for the difference between acres sown and acres inspected.)

**Conclusions:**

A lack of knowledge about the hemp life cycle greatly contributed to the failure of high CBD hemp crops in Arizona. Other problems included noncompliance with tetrahydrocannabinol limits, poor sources for seeds and inconsistent genetics of the hemp varieties sold to farmers, and diseases that hemp plants were susceptible to such as *Pythium* crown and root rot and beet curly top virus. Addressing these factors will go far in making hemp a profitable and widespread crop in Arizona. Additionally, hemp grown for other traditional uses (e.g., fiber or seed oil) as well as new applications (e.g., microgreens, hempcrete, and phytoremediation) offers other pathways for successful hemp agriculture in this state.

## Background

*Cannabis sativa* L. is a plant grown by farmers for a multitude of reasons. For medical and recreational uses, the plant is desired for the psychologically active secondary metabolites found mostly in trichomes on and around the female flowers. For industrial and commercial uses, the plant is valued for its fibers that are used in ropes, clothing, and sails, flowers for oil, and seeds for oil or food. Hemp cultivation in the USA was not legal at the federal level until the passage of the 2018 Farm Bill (U.S. Government n.d.). While growing cannabis with high levels of its main psychoactive active compound, tetrahydrocannabinol (THC), was still illegal at the federal level, growing hemp with high levels of cannabidiol (CBD), a non-psychoactive secondary metabolite, was not prohibited. With the increasing number of states that had legalized medical and/or recreational cannabis fostering demand for cannabis-related compounds, entrepreneurs attempted to profit from this interest by creating and selling CBD containing products throughout the country, in accordance with the new law. This created a perception amongst agriculturalists that they could make medical/recreational cannabis level profits, estimated to be as much as five times the revenue from an equivalent acreage of soybeans (Jibilian 2019), from growing strains of hemp that was bred for its high CBD concentrations.

The Arizona Department of Agriculture (AZDA) started an industrial hemp program in 2019 (Arizona Department of Agriculture (AZDA) 2022). Yuma — a county that provided 30% of the agricultural revenue of Arizona, USA ( U.S. Department of Agriculture (USDA) 2017) — once had over 200 farmers sowing seeds for CBD-producing hemp crops. Unfortunately, the hemp industry did not continue to grow as was hoped, and within a year only, a handful of farms still had active hemp fields. This paper will focus on hemp agriculture in Yuma County, AZ, USA, because it is the largest producer of hemp crops in Arizona and has an active agricultural extension program (represented by one of the authors, R. Masson) that has examined the use of hemp crops in the region. The objective of this paper is to (i) examine the boom and bust of high CBD hemp agriculture in Arizona, (ii) determine what factors present in the life history of the plant as well as the environmental conditions of agricultural areas in Arizona led to the rapid decline of the crop, and (iii) propose actions that might help future farmers interested in hemp crops in Arizona, USA, and beyond. We used a combination of mapping analysis using data obtained from AZDA industrial hemp program along with information collected through an extension outreach program in Yuma County to evaluate why hemp agriculture collapsed as well as to foster solutions to these problems. This analysis will facilitate and improve future attempts to grow hemp in Arizona, USA, and other parts of the world with similar growth conditions.

## Methods

### Data sources and visualization

The Arizona Department of Agriculture issues licenses for growing and producing hemp, maintaining records every year since 2019 (Arizona Department of Agriculture (AZDA) 2022). We submitted two public record requests to AZDA. The first request was for all of the hemp licenses applied for in 2019, 2020, and 2021. The data were compiled from all of the permit applications to grow hemp filed with AZDA, including (i) the locations registered for growing, harvesting, and processing hemp and (ii) the number of outdoor sites, indoor growth facilities, nursery facilities, and processing facilities at each location. The second request was for data from AZDA field inspections of all hemp farms that were actively growing the plant in 2019, 2020, and 2021. These field inspection data included the following: (i) the acreage of hemp farms inspected in each county and (ii) the number of inspections that identified THC overage (i.e., hemp containing *Δ*^9^-THC over the legal 0.3% limit by dry weight). All data were tallied by AZDA in April 2022. This study relied mainly on the field inspection data, as it contained more precise information about what was actually growing in agricultural fields in Arizona. The locations of farms were geocoded using the coordinate information contained in the public records. The data were then separated by year, and a time series of maps was assembled using ArcGIS Pro (Esri, Redlands, CA, USA) to visualize the boom and bust of hemp for of the entire state of Arizona, USA, with an emphasis on Yuma County.

### Outreach activities and survey

During the 2019, 2020, and 2021 hemp growth seasons, one of the authors (Masson) met with about 75 hemp farmers in Yuma County as a part of the University of Arizona (U of A) extension’s outreach program. Extension activities during these growing seasons included numerous talks and presentations about growing industrial hemp that produced large quantities of CBD. The talks focused on agricultural techniques and unique issues involved in growing hemp in the farms of Yuma County. We met with farmers in the 2019 and 2020 hemp grow season meetings and collected farmers’ opinions about seed sources and marketing issues. We also distributed a five-question survey to the farmers to identify their agricultural interest and educational needs. This survey was detailed and reported in another publication (Masson 2020b), and the questions used are as follows:

1. Where are you from?
2. What is your role in agriculture?
3. What media sources do you like to receive educational information from?
4. What agricultural topics would you like more educational resources provided to you from the Yuma County Cooperative Extension Department?
5. Which topics of hemp production would you like to know more about?

## Results

### A lack of experience and THC overage plague Arizona’s hemp grow from 2019 to 2020

The first hemp grow season in Arizona began with many farmers deciding to sow high CBD hemp in the summer of 2019. Out of the 227 licensed growers in Arizona (Table 1), there was a variety of people trying to enter the hemp industry. Many were inexperienced farmers new to agriculture and looking to profit from the crop. However, several unexpected issues prevented a successful first season. The following issues were mentioned frequently by local farmers in discussions with one of the authors (R. Masson), although no quantification of how often each issue affected how many acres was not possible. First, many growers were obtaining seeds from areas of the world (no hemp seed sources are currently grown in Arizona) that were located at different latitudes from Arizona, so the seeds were not adapted to the photoperiod for southern Arizona. Since southern Arizona is closer to the equator than most areas that the hemp seeds were originally grown in, the farms in Yuma have a shorter day length than what the plants were adapted to. This could cause the plants to begin flowering too early, which would greatly reduce the size of the plant and the amount of biomass available for harvest. Additionally, seed quality was variable due to the lack of regulation in the hemp seed industry, which meant there was no governing body checking the seeds. Despite the uncertain quality of seeds, many growers were found to have purchased seeds at a high price. A second issue that hindered success during the first year of cultivation was poor timing. In 2019, hemp crops were started in July (Arizona Department of Agriculture (AZDA) 2019), when the high temperature for the region reached or exceeded 43 °C (110 °F) for 9 days and averaged over 42 °C (108 °F); (AZMET 2019). This heat proved to be severely detrimental to the crop (Masson 2021). Many growers opted to use transplants instead of seeds, which turned out to respond poorly to the climate in Arizona. Transplants were rooted cuttings and were selected by some farmers who hoped to benefit from the genetic uniformity of transplants that were genetically identical. The transplants did not fair well, and this is suspected a result from the cuttings being rooted in less harsh environmental conditions than the fields they were planted in. A third issue that plagued the first growing season was an excess of *Δ*^9^-THC (beyond the 0.3% legal limit), which if not remediated would render the crop illegal and necessitate its destruction. Due to the initial boom overwhelming the ability of AZDA to perform field inspections, many fields exceeded the legal limit before they could be assessed for compliance (Table 2), causing farmers to be forced to destroy their crops. As hemp matures, it produces more THC, making it crucial that the compliance inspection be done before the THC levels exceed legal limits. At the start in 2019, there were 99 active farms growing hemp in Yuma County and the surrounding area. As the crop reached maturity, the hemp plants produced a strong and noxious odor, made even more noticeable due to the summer heat (average high temperature for July 2019, 42 °C (108 °F); August, 43 °C (110 °F); (AZMET, 2019)). This deterred a lot of farmers from trying to grow hemp in the second year. Lastly, there was a drop in the market price of hemp in the end of 2019 (Schmidt 2020; Nobles 2019), and many farmers could not find buyers before their harvest aged out. As a result, several farms in the Yuma area suffered heavy loss and bankrupted.

**Table 1:** Active hemp growth by county inspected by the Arizona Department of Agriculture in the years of 2019, 2020, and 2021

	***2019***		***2020***		***2021***	
**County**	***Number of growers***	***Acres***	***Number of growers***	***Acres***	***Number of growers***	***Acres***
**Apache**	3	3	15	18	4	25
**Cochise**	10	63	6	3	8	5
**Coconino**	1	1	7	1	3	< 1
**Graham**	9	103	18	89	2	0
**La Paz**	1	20	1	3	9	< 1
**Maricopa**	52	1126	75	331	27	8
**Mohave**	49	549	13	25	6	27
**Navajo**	5	6	13	3	12	< 1
**Pima**	2	1	39	21	14	< 1
**Pinal**	15	207	32	165	11	< 1
**Santa Cruz**	8	4	3	4	2	2
**Yavapai**	2	< 1	4	2	2	0
**Yuma**	70	1806	40	458	13	56
**Total**	227	3890	266	1123	113	128

**Table 2:** Statewide planting acreage of hemp based on the inspection records of the Arizona Department of Agriculture in the years of 2019, 2020, and 2021

**Statewide grower data**	**2019**	**2020**	**2021**
Acres planted	5430	869	156
Acres inspected near harvest	3890	1123^a^	128
Harvest lots sampled	227	290	104
Samples passed (*Δ*^9^-THC < 0.3% by dry weight)	154	153	61^b^
Samples non-compliant	73	136	69
Percentage of acres non-compliant	25%	47%	53%
Percentage of acres non-compliant after remediation or retest	20%	39%	52%

^a^Some acres planted in 2019 were not inspected until 2020

^b^Some hemp samples are retested

The second hemp grow season in Arizona, USA, was marked by a 84% decrease in the total acreage planted from 5430 acres in 2019 to 869 acres in 2020 (Table 2). During the 2020 season, the THC overage remained an important problem with 47% of the acreage exceeding the THC limit (Table 2). In 2021, only 156 acres of hemp were planted. Most of this acreage was from a single grower in Yuma, and a majority of the total hemp grown was later found to exceed the THC limit (i.e., 53% in total; Table 2).

During both the 2019 and 2020 growing seasons, two diseases were found in hemp fields in Arizona, USA, which could both significantly reduce yields and thus the economic viability of hemp as a crop. The first disease, *Pythium* crown and root rot, is a common disease of hemp that has been observed in outdoor hemp crops in Arizona, California, Indiana, and North Carolina, USA (Hu and Masson 2021a). Pythium crown and root rot are caused by several species of water molds (oomycetes) in the genus *Pythium*, such as *Pythium aphanidermatum*, *Pythium ultimum*, and *Pythium myriotylum*. Crown and root rot are a widespread disease of agricultural crops such as cabbages, carrots, melons, peppers, and corn (Hu and Masson 2021a). The second disease found in hemp fields was beet curly top virus (BCTV) (Hu et al. 2021). BCTV is a member of the Geminiviridae family that can infect numerous crops such as beans, cabbage, melons, and peppers (Hu and Masson 2021b). BCTV is spread by phloem-feeding leafhoppers such as *Circulifer tenellus* in the USA, where the insects pick up the virus while overwintering on weeds such as Russian thistle, tumble mustard, four-wing saltbush, and pigweed and then move to agricultural crops in the spring spreading the virus (Hu and Masson 2021b). While no quantitative data was taken on the extent of either of these diseases, their presence in the crop at its first few harvests in Yuma County suggests that both would be a significant problem for farmers trying to grow hemp.

### Total number of hemp farms and acreage in Arizona declined between 2019 and 2021

**Fig. 1:**
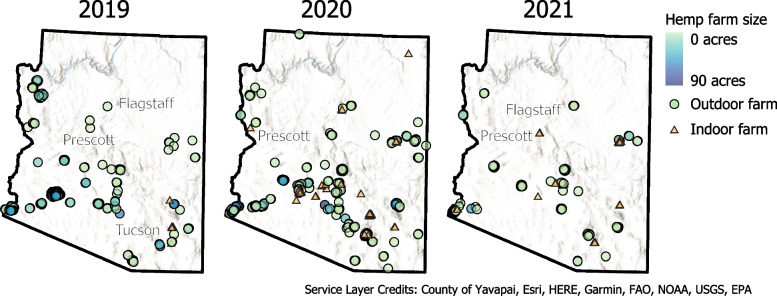
Active grows in the state of Arizona, USA, for 2019, 2020, and 2021, respectively. In 2019, there were 227 active grows in Arizona. Only eight were indoor grows. In 2020 and 2021, there were 61 and 26 indoor grows respectively. The acreage information was only available for outdoor grows, not indoor grows

Hemp farming in Yuma County occurs almost exclusively outdoors, in part due to the area’s access to water and soil fertility (Arizona Department of Agriculture (AZDA) 2018). Figure 1 shows a map of Arizona, USA, and its active hemp grow locations for 2019, 2020, and 2021. In 2019, there were 227 farms growing hemp, and this number jumped to 266 in 2020. However, by 2021, there were only 113 farms growing hemp. An even greater decline was seen in the total acreage of hemp produced declined from year to year. The first year, 2019, was the peak with 3890 acres, but this number declined by over 70% to only 1123 acres in 2020. Additionally, the total acreage declined precipitously by 89% in 2021 with only 128 acres grown in the entire state. This sharp decline is representative of a distrust in the industry as people are no longer willing to stake large parts of their land on hemp. It is consistent with the lack of enthusiasm in hemp research reported earlier in a farmer survey (Masson 2020b).

**Fig. 2:**
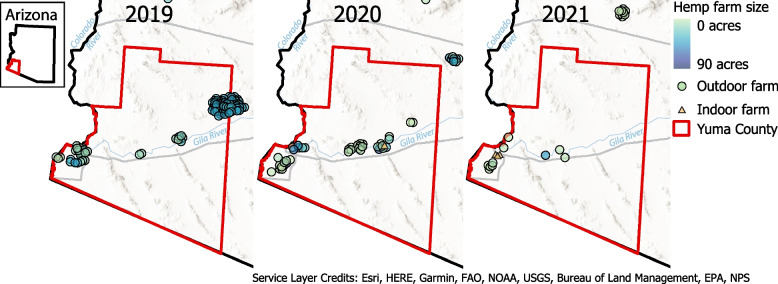
Active grows in Yuma County for 2019, 2020, and 2021, respectively. In 2019, there were 99 active grows in Yuma and surrounding areas and 70 within the Yuma County boundary. No indoor grows were in the area. In 2020 and 2021, there were 40 and 13 active growers in Yuma, with only one and three indoor grows, respectively. The acreage information was only available for outdoor grows, not indoor grows

Figure 2 indicates the boom and bust of high CBD hemp in Yuma County, AZ, USA. At the start of 2019, there were 99 active farms growing hemp in Yuma County and the surrounding area, with 70 of those in Yuma County itself. By 2020, the number of hemp farms dropped to 40, and in 2021, there were only thirteen active hemp-growing farms left in the region. Based on data for Yuma County, hemp growth as measured by the number of acres that were planted with the crop decreased 97% in 2 years in an active agricultural area (Table 1). This is a very sharp decline for a brand-new market with a lot of potential.

## Discussion

First and foremost, the failure of high CBD hemp in Arizona, USA, is a market consequence. Because of the low prices for CBD (Jibilian 2019) and limited infrastructure in Arizona, hemp does not appear to be a viable crop currently for Yuma County. The other reasons behind the failure of high CBD hemp as a crop in Arizona can be attributed to the plant’s own life history and a lack of familiarity with it on the part of the farmers. The first likely problem was that the seed farmers obtained were not bred for seed-based agricultural applications. Cannabis strains that produce high yields of CBD were first bred from high THC strains used in the indoor medical/recreational cannabis industry (Grassa et al. 2021). Small-scale greenhouse or indoor growing of THC strains primarily utilize clonal propagation or purchase of seed from established sources utilizing stable varieties. The newness of hemp and high demand for seed rushed the seed production process that could have resulted in seeds of varying quality. Previous research into the genomics of CBD producing hemp strains demonstrates that the high CBD-producing varieties are a paraphyletic group, with fiber and grain producing strains nested within the CBD clusters (Johnson and Wallace 2021). The most reasonable explanation for this is that high CBD strains have been bred repeatedly from other hemp strains and are not a group defined by a single high CBD-producing ancestral strain. This research also demonstrated that even within a single variety of CBD-producing hemp, there was large amounts of variation, with some strains having variation that scattered across the entire 173 plant phylogenetic tree (Masson 2020a).

Some of the problems that this variation can present to outdoor farming operations often involve the photoperiod of the cannabis plant. Cannabis is a short-day plant, meaning it will start to flower when the length of daylight falls below a certain amount (Hall et al. 2012). Cannabis varieties in the past had been bred to flower at various day lengths, to adapt the plant to different latitudes where growing takes place (Small 2017). Since CBD is produced in trichomes around the female flower, getting the plant to produce flowers is essential to commercial success. Having plants that flower too late or too early can greatly reduce the amount of usable inflorescence biomass for CBD extraction that the farmer is able to harvest. In Yuma County, this often meant that hemp plants would flower too early, resulting in a much smaller plant at maturity and a significant loss of inflorescence biomass available for harvest and extraction (Masson 2021).

Other problems with the plants involved the production of THC and terpenes. Plants that produce more than 0.3% *Δ*^9^-THC by dry weight are no longer considered hemp (first proposed by (Small and Cronquist 1976)) and must be remediated by mixing biomass with flowers and retested (Masson 2021). Informal estimates from communications with the farming community as well as data reported in this study revealed that about one-third of Arizona, USA’s CBD crop was destroyed each year because of THC overage. Since both CBD and THC are produced from the same precursor, olivetolic acid (Flores-Sanchez and Verpoorte 2008), their presence in hemp plants is strongly related to each other. Thus, a relaxation of the allowable THC content below 0.3%, the current legal limit, would be beneficial to the industry. Given that a 1% *Δ*^9^-THC content is widely recognized as the minimum amount to induce a noticeable intoxicating effect (Small 2018), there appears to be room to raise this limit. The original 0.3% limit proposed by Small and Cronquist (1976) was described by the authors as arbitrary and certainly has little direct support from physiological research. Also, the high CBD hemp had a strongly unpleasant odor, almost certainly from the terpenes that the plant synthesizes along with cannabinoids. These terpenes are highly valued in the medicinal/recreational cannabis industry, giving the inflorescences used for medical and recreational consumption their unique smells and flavors (Booth and Bohlmann 2019). The terpenes are usually removed in the process of extracting and purifying CBD, but their presence in the crop was a real deterrent for the farmers and their neighbors.

While there were many failures during the boom and bust of hemp in Arizona, USA, there are some glimmers of hope. The U of A outreach program was able to identify three successful growers in Yuma. One was a small-scale grower. A second farmer mostly profited on agro-tourism, hosting parties, and providing a novel “pick-your-own hemp” roadside stop. A third farm was managed intensively to harvest before the crop exceeded the legal THC limit. These cases demonstrate that there can be success growing hemp for CBD in Arizona, USA. However, the bust has already driven many potential farmers away from hemp as a crop. While growing for CBD may be an unrevivable industry, there is still room for lower CBD hemp to be successful when grown for fiber or other uses. Additionally, hemp may provide a value-added option to remove environmental contaminants as a new phytoremediation crop in Arizona, USA (Wu et al. 2021). Phytoremediation is the use of plants to clean up soil or water from toxic contaminants such as heavy metals or organic pollutants. The Arizona Department of Agriculture has recently taken several steps to support hemp agriculture, such as allowing farmers to be eligible for federal programs including the Noninsured Crop Disaster Assistance Program and Easement Program Farm loans, as well as allowing the export to other states of hemp nursery products including seedlings and rooted cuttings ( Arizona Department of Agriculture (AZDA) 2022).

At the time of writing, only ca. 60 acres of hemp grow exists in Arizona, USA. Most of the hemp products being sold in Arizona, USA’s market are not from hemp grown in the state. Future production in hemp can utilize varieties that have been selected for different parts of the plant that have commercial value, such as high-fiber or seed production. Currently, varieties are well established that can be grown for fiber or seed (used for seed oil or as a food or feed crop). Selecting strains that would be successful in the climate of Yuma County should start with varieties bred for similar climates in other regions of the world. Leafy greens, or microgreens, are leaves harvested from young plants and are currently prized by consumers for their nutritional qualities and flavor (Martínez-Sánchez et al. 2012). Recently, the use of hemp to grow microgreens has also been developed (Mi et al. 2020) and represents a large and untapped potential for hemp agriculture. Nontraditional products can also be made with hemp, such as hempcrete. Hempcrete is a form of concrete that is made with hemp shives, the part of the stem not used for fiber production. Hempcrete has been found to have better sound insulation properties and an ability to regulate relative humidity inside of buildings (Lupu et al. 2022), as well as being a potential carbon sink to help reduce the causes of climate change (Kumar et al. 2021).

Any discussion of sustainable agriculture in Arizona, USA, must also consider the current state of water resources. Arizona is an arid state, and water for agriculture is provided from surface waters such as the Colorado River in the case of Yuma County. Colorado River water is a disappearing resource, and recently, the federal government has asked the states of the Colorado basin to revise their allotted amounts of river water based on the decline of reservoirs in the system due to climate change (Vasilogambros 2022). Hemp for fiber has been suggested as an alternative to water intensive crops such as cotton, and some research claims that it uses half as much water as the latter (Rosa and Grammatikos 2019), though this was not the objective of the cited study. Research into water usage in hemp grown in southern Italy demonstrated that bisexual varieties cultivated for fiber used 250-mm (almost 10 inch) water, and dioecious varieties also bred for fiber used 450 mm (almost 18 inch) (Cosentino et al. 2013). Research on water use in hemp crops grown in Tasmania found it possible to grow the crop with between 359 mm (14 inch) and 535 mm (21 inch) of water, although significant yield differences were found between different water levels (Lisson and Mendham 1998). Other research conducted in Spain demonstrated the ability to grow hemp as a fiber crop with 960 mm (38 inch) up to 1200 mm (47 inch) of water, again showing significant differences in the yields for different irrigation levels (García-Tejero et al. 2014). Research in Arizona has noted that cotton crops grown in Maricopa County consume 1050 mm (41.2 inch) of water for a growing season (Erie et al. 1965). While this number is higher than most of the amounts used by hemp cited, it is not greater than all of them, suggesting that hemp may not be the water-saving crop some have claimed. Furthermore, none of the hemp studies cited above was conducted in desert environments similar to Arizona or with hemp varieties that are grown for high CBD production. Given the lack of suitable comparisons, any claims of water conservation from replacing cotton (or any other crop) with hemp are speculative at best until supported with research from arid environment agricultural systems.

## Conclusions

Hemp agriculture in Arizona, USA, in terms of a crop that produces significant amounts of CBD, would seem to have failed based on the boom and bust cycle of the 2019, 2020, and 2021 grow seasons. This trend mirrors those seen across the USA (Alpert 2020), and the reasons that it occurred in Arizona should be helpful in understanding the phenomenon at the national level. For hemp agriculture to be successful, certain steps must be taken to avoid the problems discussed here. Certainly, the first step would be to breed a true breeding strain that is adapted to the photoperiod of the latitude at which it is to be grown. The strains should also be selected to not produce THC, or at least adapted to growing conditions in Arizona, so that THC production does not exceed the legal limit, as this was a major problem for growers. While such a strain might not be possible from conventional breeding techniques, genetic engineering that deactivates the THC synthase genes might also be considered to create a THC-free high CBD strain suitable for outdoor agriculture. Work on developing a THC-free high CBD strain is currently under way in the private sector; however, no seeds of such a strain are currently available to farmers.

Other approaches to making hemp successful in Arizona, USA, could be developed, including novel applications such as microgreens for human consumption or for other products such as hempcrete. Traditional hemp-based crops could also be considered, such as seed oil or fiber. Any of these approaches will have to take into consideration the limits unique to Arizona agriculture, especially the climate and availability of water. With climate change making water scarcer, and the human population increasing, it would be prudent to assess alternative crops, including the many uses of hemp, in creating sustainable agriculture in Arizona for the twenty-first century and beyond.

## Data Availability

The data and preliminary analysis of this study are available upon request.

## Abbreviations

AZDA: Arizona Department of Agriculture
BCTV: Beet curly top virus
CBD: Cannabidiol
RNA: Ribonucleic acid
THC: Tetrahydrocannabinol
USDA: United States Department of Agriculture
Mm: Millimeters
